# Development of Recombinant Oncolytic rVSV-mIL12-mGMCSF for Cancer Immunotherapy

**DOI:** 10.3390/ijms25010211

**Published:** 2023-12-22

**Authors:** Anastasia Ryapolova, Ekaterina Minskaia, Nizami Gasanov, Vasiliy Moroz, Bogdan Krapivin, Alexander D. Egorov, Victor Laktyushkin, Sofia Zhuravleva, Maksim Nagornych, Elena Subcheva, Alexander Malogolovkin, Roman Ivanov, Alexander Karabelsky

**Affiliations:** 1 Department of Gene Therapy, Sirius University of Science and Technology, Olympic Avenue, 1, 354340 Sochi, Russia; ryapolova.av@talantiuspeh.ru (A.R.); gasanov.nb@talantiuspeh.ru (N.G.); moroz.vd@talantiuspeh.ru (V.M.); krapivin.bn@talantiuspeh.ru (B.K.); egorov.ad@talantiuspeh.ru (A.D.E.); laktyushkin.vs@talantiuspeh.ru (V.L.); zhuravlyova.sv@talantiuspeh.ru (S.Z.); nagornyh.mo@talantiuspeh.ru (M.N.); subcheva.en@talantiuspeh.ru (E.S.); ivanov.ra@talantiuspeh.ru (R.I.); karabelskiy.av@talantiuspeh.ru (A.K.); 2 Department of Molecular Virology, First Moscow State Medical University (Sechenov University), 20 Pirogovskaya, 119991 Moscow, Russia; malogolovkin.as@talantiuspeh.ru

**Keywords:** oncolytic viruses (OV), vesicular stomatitis virus (VSV), anti-cancer therapy, oncology, melanoma

## Abstract

Anti-cancer therapy based on oncolytic viruses (OVs) is a targeted approach that takes advantage of OVs’ ability to selectively infect and replicate in tumor cells, activate the host immune response, and destroy malignant cells over healthy ones. Vesicular stomatitis virus (VSV) is known for its wide range of advantages: a lack of pre-existing immunity, a genome that is easily amenable to manipulation, and rapid growth to high titers in a broad range of cell lines, to name a few. VSV-induced tumor immunity can be enhanced by the delivery of immunostimulatory cytokines. The targeted cytokine delivery to tumors avoids the significant toxicity associated with systemic delivery while also boosting the immune response. To demonstrate this enhanced effect on both tumor growth and survival, a novel recombinant VSV (rVSV)-mIL12-mGMCSF, co-expressing mouse IL-12 (interleukin-12) and GM-CSF (granulocyte-macrophage colony-stimulating factor), was tested alongside rVSV-dM51-GFP (rVSV-GFP) that was injected intratumorally in a syngeneic in vivo C57BL/6 mouse model infused subcutaneously with B16-F10 melanoma cells. The pilot study tested the effect of two viral injections 4 days apart and demonstrated that treatment with the two rVSVs resulted in partial inhibition of tumor growth (TGII of around 40%) and an increased survival rate in animals from the treatment groups. The effect of the two VSVs on immune cell populations will be investigated in future in vivo studies with an optimized experimental design with multiple higher viral doses, as a lack of this information presents a limitation of this study.

## 1. Introduction

Oncolytic viruses (OVs), consisting of diverse families of viruses, are a relatively novel, alternative class of anti-tumor therapies [1] which has progressed due to the advances in recombinant DNA technology that provided the essential tools for understanding the intricate details of virus biology. Due to the innate tropism of some viruses for tumor cells and the dysfunction of factors that disrupt viral proliferation and increase the rate of viral clearance in tumor cells (interferon type 1 response genes, tumor suppressor proteins, etc.), OVs are able to selectively replicate in malignant cells and activate the host immune response [2]. This leads to the death of malignant cells only, setting OV-based therapies apart from non-specific conventional chemo-/radiotherapies and even target-specific antibody-based therapies [3]. Recombinant OVs can be additionally modified by directed evolution to increase viral tropism for cancerous cells and reduce tropism for healthy cells. Genetic engineering of viral genomes allows for the development of safe and powerful tumor-specific viruses that also express cytotoxic, immunomodulatory, or proapoptotic genes [4].

Interferon type 1 (IFN-α), tumor necrosis factor alpha (TNF-α), interleukin-2 (IL-2), and other cytokines are known for their powerful immunostimulatory abilities [5]. Depending on the type of cytokine, the expression of these genes leads to phagocytosis, the activation of a tumor-specific innate immune response, and the activation of CD4^+^ and CD8^+^ T lymphocytes and natural killer (NK) cells. However, their systemic administration often leads to severe side effects, limiting their use [6,7,8,9]. Although systemic administration of IL-12 has been associated with serious side effects in a number of clinical studies, its targeted delivery by OVs can overcome this hurdle while, at the same time, taking full advantage of IL-12’s ability to activate both innate and adaptive immunity, as it induces T-helper 1 (Th1) differentiation and activates IFN-γ production in NK, CD4^+^, and CD8^+^ T lymphocytes [10,11,12,13]. GM-CSF can stimulate potent anti-tumor responses by promoting the maturation and activation of cross-presenting dendritic cells (DCs) [14]. For example, intratumoral injection of the vaccinia virus encoding GM-CSF and the oncotoxic protein lactaptin demonstrated inhibition of xenograft growth and glioblastoma metastases [15].

While only several OV-based therapies have been registered in the world so far (RIGVIR™, Oncorine™, Imlygic™, and Adstiladrin), the number of OV-based clinical trials is steadily growing and have exceeded one hundred (www.clinicaltrials.gov, accessed on 10 February 2023), and there is every reason to expect the emergence of new, more effective OV-based therapies [16,17]. For instance, a combination of OVs with various checkpoint inhibitors demonstrated high therapeutic potential for the treatment of resistant, inoperable, or recurrent forms of cancer [3]. A great overall response (90% and 60% complete response) to the treatment of inoperable stage 3–4 melanoma with Imlygic^®^ (talimogene laherparepvec, Amgen) was observed in combination with pembrolizumab, nivolumab, and ipilimumab [18]. As a result of this success, Imlygic^®^, the first HSV1-based therapy delivering the human GM-CSF gene, was approved for the treatment of melanoma in the USA in 2015. Its efficacy is currently being tested for the treatment of ovarian, pancreatic, rectal, and other cancers.

VSV is a negative-strand RNA virus that belongs to the family *Rhabdoviridae* and encodes only five genes (N, P, M, G, and L). Nucleocapsid (N) and large (L) proteins are essential for transcription and replication; phosphoproteins (P) for polymerase activity; matrix protein (M) for virus packaging and inhibition of the IFN response; and glycoprotein (G) for cell entry. The virus offers a promising platform for OV development, especially in combination with immunotherapeutic approaches [19]. While accidental cases of VSV infections have been reported previously, they are mild and asymptomatic, which demonstrates the overall safety of this platform [20]. The lack of pre-existing immunity, easily manipulated genome, independence on the cell cycle, and rapid growth to high titers in various cell lines make VSV an attractive platform for targeting malignant cells [21,22].

Melanoma, the most lethal type of cancer worldwide, has a high rate of metastasis at a later stage, despite its early detection [23]. Approximately 40–65% of melanoma patients do not respond to checkpoint inhibitor treatments, and the underlying mechanisms are not well understood [24,25,26]. Thirty OV-based clinical trials are evaluating treatments against melanoma in over 1000 patients. OVs can reach organs such as the brain, the common site of metastasis, cause immunogenic death of tumor cells, lead to the release of tumor antigens, and create a pro-inflammatory environment, thus enhancing the efficacy of checkpoint inhibitors. The B16-F10 murine model is a common experimental model with a long history of usage and metastatic potential [27,28].

The aim of this study was to investigate the effect of two rVSVs, expressing either green fluorescent protein (GFP) or the immunomodulatory mIL12-mGMCSF fusion protein, on both survival and tumor growth in syngeneic C57BL/6 mice subcutaneously injected with murine melanoma B16-F10 cells. While the GFP-delivering rVSV was tested for its oncolytic abilities only, the mIL12-mGMCSF-armored version was expected to achieve a heightened synergistic effect, resulting in higher inhibition of tumor growth. As the success of in vivo studies depends on the optimal experimental design, this pilot study tested the effect of two viral injections 4 days apart for an initial assessment of rVSV activity.

## 2. Results

### 2.1. Production and Characterization of rVSV-GFP and rVSV-mIL12-mGMCSF

**Figure 1:**
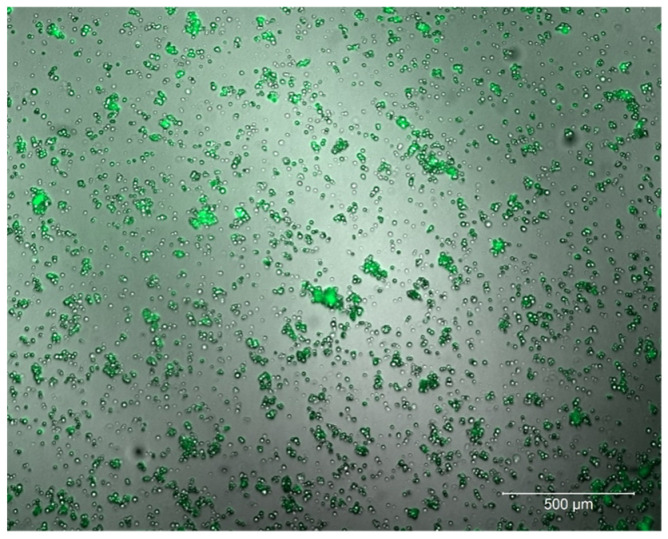
Recovery of rVSV-GFP. CPE and GFP fluorescence were observed in HEK293TN cells at 24 h post-transduction.

The two important goals of this study were to create a simplified helper virus-free method for rVSV production and to produce a sufficient number of active rVSV viral particles. Recombinant *Vaccinia virus* (rVV) used for the expression of bacteriophage T7 polymerase was substituted with a T7 polymerase-expressing plasmid. Therefore, the primary assembly of replication-competent rVSV-GFP, expressing the five VSV proteins together with the GFP reporter, was produced according to the previously reported modified protocol [29] by co-transfection of the plasmids expressing the four VSV proteins (VSV-P, VSV-G, VSV-N, and VSV-M) under the control of the cytomegalovirus immediate-early enhancer/chicken β-actin (CAG) promoter, pCAG-T7pol, and pVSV-dM51-GFP into HEK293TN cells. pVSV-dM51-GFP, also called “the core plasmid”, encodes the antigenome-sense (positive-sense) VSV RNA sequence, which serves as the template for viral genome replication. The cytopathic effect (CPE) and GFP expression were observed 72 h post-transfection and 24 h after supernatant infection (Figure 1).

**Figure 2:**
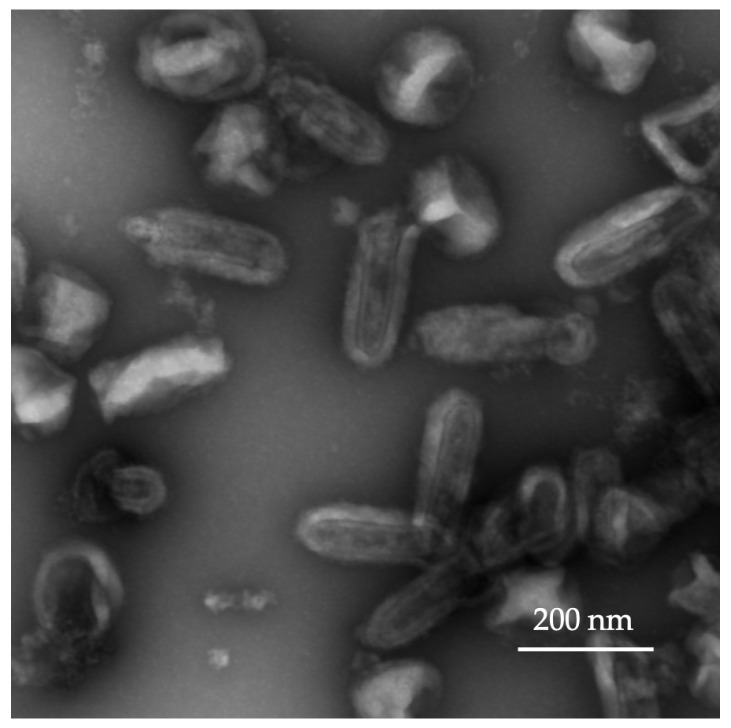
Morphologic characterization of rVSV-GFP by TEM.

The amplification of rVSV-GFP in BHK-21 cells involved transduction (infection) with post-transfection-conditioned culture media containing assembled virus particles. Three rounds of virus amplification were carried out for the described experiments; however, the number of production steps usually depends on the amount of virus required for in vitro or in vivo experiments. The virus titer was assessed by CPE in BHK-21 cells 72 h post-infection. The rVSV-GFP titer (TCID50/mL) was calculated according to the Reed–Muench method and was 3 × 10^9^ TCID50 in a 30 mL volume. For intratumoral injections at the desired dose, no further purification of rVSV-GFP was carried out, and the virus preparation was diluted to the final dose (1 × 10^6^ and 1 × 10^8^ TCID) in a 50 µL volume. The rVSV-GFP preparation was analyzed using transmission electron microscopy (TEM) (Figure 2). Bullet-shaped virions with an interior striated pattern and lipid bilayer, consistent with the features of an intact VSV virus, were observed.

**Figure 3:**
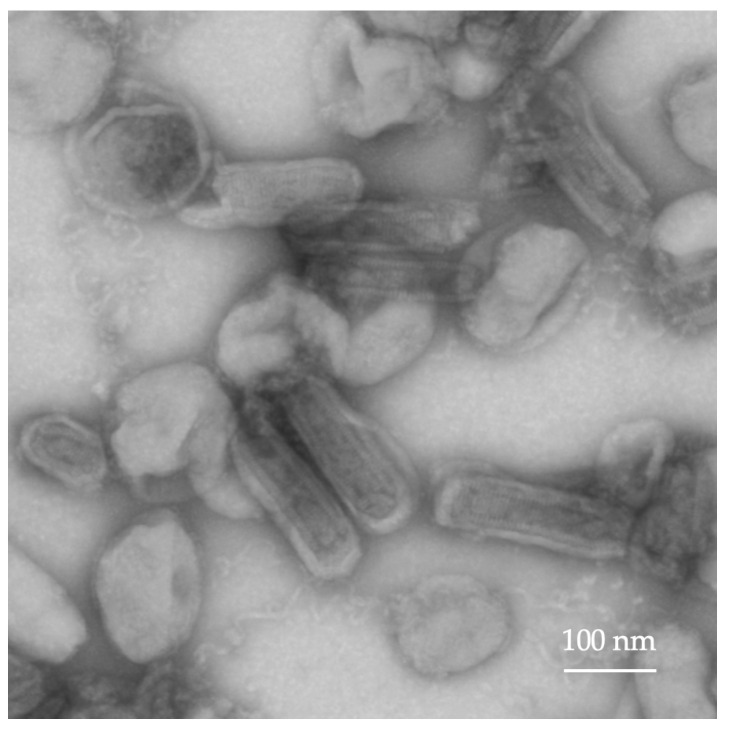
Morphologic characterization of rVSV-mIL12-mGMCSF by TEM.

The production of replication-competent rVSV-mIL12-mGMCSF, delivering the mouse IL12-GMCSF fusion, was carried out as described above for rVSV-GFP. CPE was observed 72 h post-transfection and 24 h after the supernatant infection of BHK-21 cells. The concentration of the rVSV-mIL12-mGMCSF preparation was 3 × 10^9^ TCID50/mL. For the second in vivo experiment, the purification of rVSV-mIL12-mGMCSF was carried out by ultracentrifugation with a sucrose cushion. The viral preparation was diluted to a final concentration of 1 × 10^7^ TCID50 in a 50 µL volume. The rVSV-mIL12-mGMCSF was analyzed using TEM (Figure 3).

**Figure 4:**
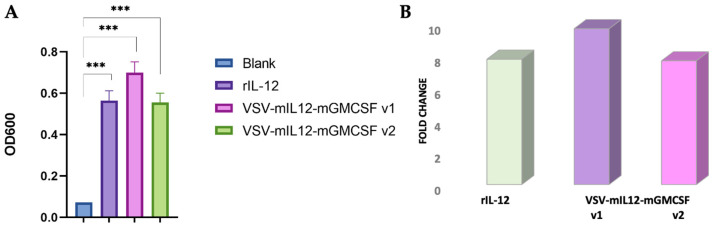
Assessment of IL-12 activity in HEK-Blue IL-12 cells. (**A**) IL-12 activity in supernatants containing rVSV-mIL12-mGMCSF amplified in two different volumes, T25 (v1) and T175 (v2) flasks, was assessed in HEK-Blue IL-12 cells by quantifying the expression levels of SEAP. SEAP expression levels were compared to the positive control, recombinant IL-12 (rIL-12), used at 10 ng/mL. Absorbance was measured on a ClarioStar plate reader at 650 nm. The statistical analysis was carried out using ordinary one-way ANOVA and Sidak’s multiple comparison test in GraphPad Prism (*** *p* ≤ 0.001). (**B**) Fold change induction of SEAP expression was compared to the blank negative control.

To confirm the activity of IL-12, which was expected to be present in rVSV-mIL12-mGMCSF supernatants, the HEK-Blue™ IL-12 reporter line, stably expressing IL-12 receptor and signaling pathway genes as well as the STAT4 transcription factor-inducible SEAP (secreted alkaline phosphatase) reporter gene, was used. As the SEAP gene is under the control of a minimal promoter, its expression only occurs in response to IL-12 stimulation, which triggers a signaling cascade leading to STAT-4 activation followed by SEAP production. The optical density (OD600) values of the samples containing rVSV-mIL12-mGMCSF supernatants from two amplification volumes were comparable (v2) or exceeded (v1) those of the positive control rIL-12 used at 10 ng/mL (Figure 4A) and were 10 (v1) and 8 (v2) times higher compared to the blank negative control (Figure 4B).

### 2.2. Intratumoral Injection of rVSV-GFP Suppresses Tumor Growth and Enhances Survival

**Figure 5:**
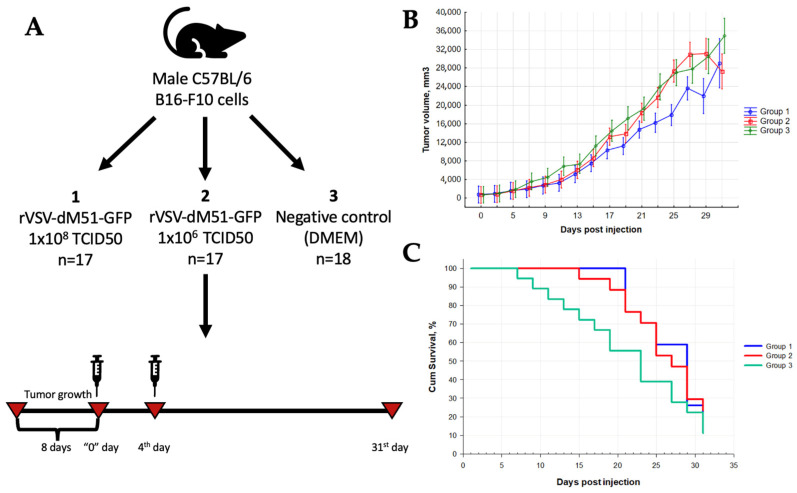
rVSV-GFP prolongs the survival of mice with murine melanoma. (**A**) Study design. (**B**) Partially inhibited tumor growth was observed in groups 1 and 2 mice compared to group 3 mice (max 52.5% and 41.8%, respectively, on day 11). The TGII in group 1 remained positive at 20–30% until the end of the study, but fell to zero in group 2 by day 25. There were statistically significant differences in tumor volumes between groups 1 and 3 for 12 days during the study (Mann–Whitney U-test, *p* < 0.05). (**C**) The increase in the median survival of the mice in group 1 was 6.1 days compared to those in group 3 (Kaplan–Meier curve).

Next, C57BL/6 mice were subcutaneously transplanted with 1 × 10^6^ B16-F10 cells, and the tumor sizes were measured every 2 days for 39 days (Figure 5A). The treatment with rVSV-GFP commenced on day 8 post-infusion, when the average tumor size reached ~500 mm^3^. Two rVSV-GFP injections 3 days apart were delivered intratumorally to group 1 (1 × 10^8^ TCID50) and group 2 (1 × 10^6^ TCID50) mice. Placebo control mice (group 3) received Dulbecco’s Modified Eagle’s Minimal Medium (DMEM) only. Overall, rapid tumor growth was observed in all three groups due to the aggressive nature of B16-F10-induced melanoma, but this process in placebo group 3 was faster (Figure 5B). Partially inhibited tumor growth was observed in groups 1 and 2 mice compared to group 3 mice (max 52.5% and 41.8%, respectively, on day 11). The TGII in group 1 remained positive at 20–30% until the end of the study, but fell to zero in group 2 by day 25 (Figure 5C). There were statistically significant differences in tumor volumes between groups 1 and 3 for 12 days during the study (Mann–Whitney U-test, *p* < 0.05). Overall, the morbidity of mice in group 3 exceeded that of the mice in groups 1 and 2. The increase in the median survival of the mice in group 1 was 6.1 days compared to those in group 3 (Kaplan–Meier survival analysis).

**Figure 6:**
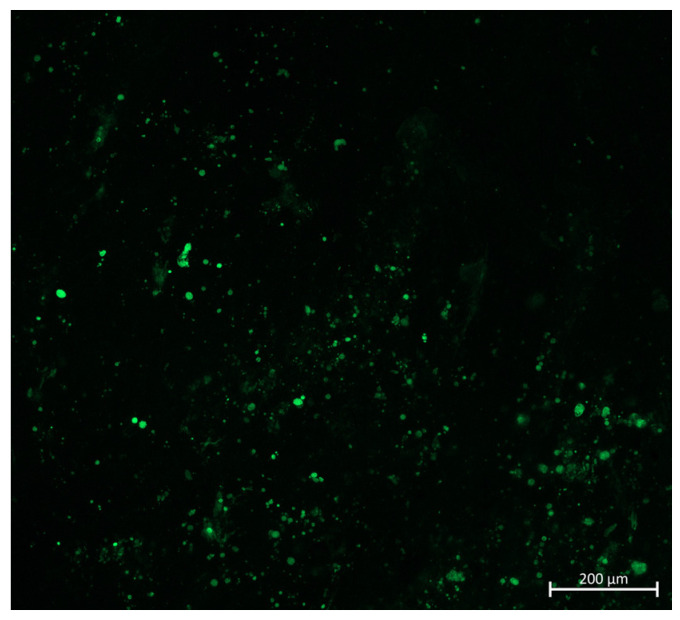
rVSV-GFP persistence in malignant nodules of mice after 21 days. Confocal fluorescence images were obtained by using an inverted point scanning confocal microscope (LSM 980 Airyscan, Zeiss).

To confirm the rVSV-GFP persistence in the tumor, one malignant nodule was extracted from group 1 mice at day 21 post-first treatment, and GFP fluorescence in the B16-F10-induced tumor was confirmed by confocal microscopy (Figure 6). These results indicate that rVSV-GFP efficiently infected and persisted in malignant cells for over 20 days.

### 2.3. Intratumoral Injection of rVSV-mIL12-mGMCSF Suppresses Tumor Growth and Enhances Survival

Following confirmation of rVSV-GFP’s oncolytic efficiency, the synergistic effect of rVSV delivering mIL12-mGMCSF (rVSV-mIL12-mGMCSF) was tested.

**Figure 7:**
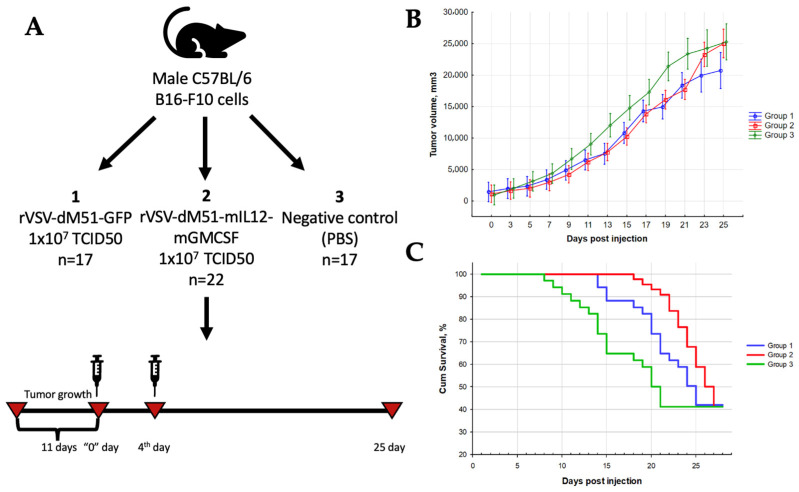
rVSV-mIL12-mGMCSF prolongs the survival of mice with murine melanoma. (**A**) Study design. (**B**) Partially inhibited tumor growth was observed in groups 1 and 2 mice compared to group 3 mice (max 37.4% and 36.9%, respectively, on days 13 and 9). The TGII in group 2 remained statistically significant until day 16 of the study (Mann–Whitney U-test, *p* < 0.05). There were statistically significant differences in tumor volumes between groups 1 and 3 for 12 days during the study (Mann–Whitney U-test, *p* < 0.05). (**C**). The increase in the median survival of mice in the treatment groups.

Similar to the first in vivo experiment, male C57BL/6 mice were subcutaneously infused with 1 × 10^6^ B16-F10 cells, and the tumor sizes were measured every 2 days for 36 days (Figure 7A). The treatment with rVSV-GFP and rVSV-mIL12-mGMCSF commenced on day 11 post-infusion, when the average tumor size reached ~500 mm^3^. Two rVSV injections (1 × 10^7^ TCID50 each) 3 days apart were delivered intratumorally to group 1 (rVSV-GFP) and group 2 (rVSV-mIL12-mGMCSF) mice. Placebo control mice (group 3) received phosphate–saline buffer (PBS) only. Rapid tumor growth was observed in all three groups, but the process in the placebo group was faster (Figure 7B). Partially inhibited tumor growth was observed in groups 1 and 2 mice compared to group 3 mice (max 37.4% and 36.9%, respectively, on days 13 and 9). The TGII in group 2 remained statistically significant until day 16 of the study (Mann–Whitney U-test, *p* < 0.05). There were statistically significant differences in tumor volumes between groups 1 and 3 for 12 days during the study (Mann–Whitney U-test, *p* < 0.05). Overall, the morbidity of mice in group 3 exceeded that of the mice in groups 1 and 2 (Kaplan–Meier survival analysis) (Figure 7C).

## 3. Discussion

It is estimated that over the next fifteen years, the number of newly diagnosed cancer cases per year will rise to 30 million and the number of cancer-related deaths to 16.4 million [30]. Unfortunately, once cancer development reaches the metastatic stage, the outlook changes drastically and the prognosis becomes poor. Cancer cells are able to avoid detection and destruction by the immune system either by expressing ligands of immune checkpoint molecules that suppress the activity of cytotoxic T cells (for example, PD-L1) or by attracting immunosuppressive cells, such as Tregs or M2 macrophages, to the tumor microenvironment [31]. Both immunotherapy, which aims to resolve these problems, and other novel therapeutic strategies present the promise of improving the survival rates of cancer patients.

OVs specifically replicate in and lyse malignant cells, a process that releases their contents, including tumor antigens, into their surroundings and activates the host immune response [32,33]. OV-induced tumor immunity can be enhanced by the delivery of anti-tumor cytokines, such as IFNα, TNFα, IL-2, IL-12, and GM-CSF, while tumor-targeted cytokine delivery avoids the significant toxicity associated with systemic delivery while also boosting the immune response.

In this study, two rVSVs, rVSV-GFP and rVSV-mIL12-mGMCSF, were successfully produced, and the quality of the viral particles was assessed by TEM (Figure 2). The functional activity of the IL-12 produced in rVSV-mIL12-mGMCSF supernatants of BHK-21 cells was assessed in the HEK-Blue™ IL-12 reporter cell line expressing the STAT4-inducible SEAP reporter gene. As shown in Figure 4B, the levels of SEAP reporter expression induced by the rVSV-produced IL-12 were up to 10 times higher than those in virus-free supernatants and comparable to those induced by the rIL-12 positive control used at 10 ng/mL.

First, the oncolytic activity of rVSV-GFP was tested in a syngeneic in vivo C57BL/6 mouse model subcutaneously infused with B16-F10 melanoma cells. As shown in Figure 5, two intratumoral injections of rVSV-GFP at doses of 1 × 10^6^ (group 1) or 1 × 10^8^ (group 2) TCID at days 8 and 11 post-inoculation resulted in 52.5% and 41.8%tumor growth inhibition, respectively, at day 11 post-virus injection. The TGII in group 1 remained positive at 20–30% until the end of the study. There were statistically significant differences in tumor volumes between groups 1 and 3 for 12 days during the study (Mann–Whitney U-test, *p* < 0.05), and the morbidity of mice in group 3 exceeded that of the mice in the treatment groups. The increase in the median survival of mice in group 1 was 6.1 days compared to group 3. Importantly, virus persistence in melanoma cells for at least 21 days was demonstrated by the confocal microscopy analysis of tumor tissues (Figure 6).

Next, the synergistic effect of rVSV delivering mIL12-mGMCSF was assessed in comparison to rVSV-GFP. When the average tumor size reached ~500 mm^3^ (day 11 post B16-F10 injection), the mice received two doses (1 × 10^7^ TCID50 each) of rVSV-GFP (group 1), rVSV-mIL12-mGMCSF (group 2), or PBS (group 3). The rapid tumor growth in group 3 far exceeded that of groups 1 and 2 (Figure 7B), with partially inhibited tumor growth evident in group 1 and 2 mice (max 37.4% and 36.9%, respectively, on days 13 and 9). The TGII in group 2 remained statistically significant until day 16 of the study (Mann–Whitney U-test, *p* < 0.05). There were statistically significant differences in tumor volumes between groups 1 and 3 for 12 days during the study (Mann–Whitney U-test, *p* < 0.05). Overall, the morbidity of group 3 mice exceeded that of the mice in groups 1 and 2 (Kaplan–Meier survival analysis) (Figure 7C). Interestingly, while the tumor growth in groups 1 and 2 did not differ, the survival rate in group 2 mice exceeded that of group 1 mice, which could be attributed to the delivery of the immunostimulatory IL-12 and GMCSF genes.

The successful outcome of in vivo experiments greatly depends on the optimal experimental design, which includes several crucial components: tumor size on the day of the first treatment, viral dose, and the number of treatments. These vary greatly from study to study and need to be optimized for improved results (higher inhibition of tumor growth and survival rate). For example, it was demonstrated that tumor growth in C57BL/6 mice was delayed, but no tumor regression was noted upon intratumoral injections of rVSV-mIFNb at 1 × 10^9^ TCID50. The virus was injected four times (two weekly doses) once the B16-F10 tumors reached 200 mm^3^. The median survival of 23 days was prolonged compared to the placebo control [34]. In another study [35], significant tumor regression (*p* < 0.05) was observed in C57BL/6 mice that were injected with a more immunogenic B16-OVA melanoma model (due to the presence of the OVA protein) [36,37]. Two weekly doses of VSV-mIFNb at 5 × 10^8^ PFU were injected intratumorally when the tumors reached 200 mm^3^. One intravenous dose of 1 × 10^8^ TCID50 and 5 × 10^7^ TCID50 was administered to C57BL/RaLwRij mice to treat myeloma in the 5TGM1 model [38]. One intravenous injection of 1 × 10^6^, 1 × 10^7^, or 1 × 10^8^ TCID50 was administered to C57BL/6j mice to treat C1498 cell-based acute myeloid leukemia in combination with anti-PD-L1 Ab [39]. This approach enhanced the survival of mice in the treatment group. Intratumoral injections of recombinant vaccinia virus (rVV) that lacked thymidine kinase and vaccinia growth factor genes but co-expressed miPDL1-mGMCSF resulted in more significant B16-F10 tumor growth inhibition compared to rVV-RFP or rVV-GMCSF [11], as demonstrated by both bioluminescence monitoring and caliper measurements. The rVV was injected three times on days 0, 3, and 7 at 5 × 10^7^ pfu/tumor.

This study demonstrated that treatment with both rVSV-GFP and rVSV-mIL12-mGMCSF resulted in a reduction in tumor growth and increased survival rate of animals in the treatment groups. The TGII in the described in vivo experiments was about 40 percent, in contrast to the 70 percent commonly required for the initiation of clinical development. We opted for two injections of rVSV-GFP and rVSV-mIL12-mGMCSF at 1 × 10^7^ TCID50 four days apart following the initial in vivo experiment comparing the effect of two doses (1 × 10^6^ and 1 × 10^8^) of rVSV-GFP on tumor growth and survival rate. Intratumoral administration was chosen because it is the most common route used in OV-based clinical trials [40]. It is also important to mention that the treatment with the two rVSVs commenced when the average tumor size reached ~500 mm^3^, which was more than double the size compared to other studies mentioned above. It is plausible to suggest that the long-term success of this therapy will require an adjustment (increase) of the viral doses, multiple treatments, and a comparison of delivery routes to achieve the desired effect, especially considering the aggressive nature of the melanoma model. Taking these adjustments into account, we hope to achieve a higher efficacy and plan to compare the effect of IL-12 and GMCSF monomers vs. the IL12-GMCSF fusion protein and to decipher the contribution of these cytokines to the anti-tumor immune response. Therefore, further in vivo studies with an optimized experimental design and multiple higher viral doses will be conduction to investigate the impact of rVSV itself and the IL12-GMCSF fusion on immune cell populations, as a lack of this information presents a major limitation of this study.

## 4. Materials and Methods

### 4.1. Cell Lines

BHK-21, HEK293TN, and B16-F10 cell lines, obtained from American Type Cell Culture, were maintained in Dulbecco’s Modified Eagle’s Minimal Medium (DMEM), 4.5 g/L glucose, supplemented with heat-inactivated 10% fetal bovine serum (FBS). HEK-Blue IL-12 cells (hkb-Il12, InvivoGen, San Diego, CA, USA) were maintained in DMEM, 4.5 g/L glucose, 2 mM L-glutamine, 10% FBS, and 100 μg/mL Normocin™ in a humidified incubator at 37 °C and 5% CO_2_.

### 4.2. Construction of Plasmids

The pVSV-dG-GFP plasmid (EH1003, Kerafast, Boston, MA, USA) was used as a vector for generating the pVSV-G-GFP and pVSV-dM51-GFP plasmids. To achieve this goal, the G gene sequence, encoding the VSV envelope protein, was PCR amplified with forward (prim#1, Supplementary Table S1) and reverse (prim#2) primers incorporating NheI and MluI restriction sites, respectively, using plasmid pBS-G (EH1016, Kerafast, Boston, MA, USA) as a template and cloned into the pVSV-dG-GFP plasmid. The resultant pVSV-G-GFP vector encoded five viral proteins together with GFP. pVSV-dM51-GFP was generated by deletion of the methionine (ATG) in position 51 of pVSV-G-GFP with mutagenesis primers (prim#3 and prim#4) (Supplementary Figure S1 and Table S1). To construct pVSV-dM51-mIL12-mGMCSF, the mIL12-mGMCSF fusion, synthesized commercially (Evrogen, Moscow, Russia), was amplified with forward (prim#11) and reverse (prim#12) primers and cloned into the pVSV-dM51-GFP plasmid in place of the GFP gene via NheI and AvrII restriction sites (Supplementary Figure S2). Plasmid pCAG-T7pol, expressing T7 RNA polymerase (T7RNAP), was purchased from Addgene (cat. No. 59926). The recovery of infectious VSV in the absence of vaccinia virus relied on pCAG-T7pol and helper plasmids expressing L (pBS-L), P (pBS-P), and N (pBS-N) genes under the control of the CAG promoter. The three genes were cloned into the pCAG-T7pol vector in place of the T7 polymerase gene via EcoRI and NotI restriction sites through PCR amplification using VSV-N, VSV-P, and VSV-L (EH1004, Kerafast, Boston, MA, USA) (as templates) and the corresponding primers (L gene: prim#5 and prim#6; P gene: prim#7 and prim#8; N gene: prim#9 and prim#10, Supplementary Table S1). All plasmids were verified by DNA sequencing.

### 4.3. Rescue and Purification of Recombinant VSV

The rescue of rVSV-GFP was carried out as previously reported [19] with one exception: T7 RNA polymerase was delivered by pCAG-T7pol plasmid transfection and not rVV. Briefly, HEK293TN cells were grown overnight in a 12-well plate to 70–80% confluency. The next day, the cells were transfected with the following plasmids: pCAG-T7pol, pCAG-P, pCAG-L, pCAG-N, pCAG-G, and pVSV-dM51-GFP at a 5:5:1:3:4:5 ratio with polyethylenimine (PEI). After 12 h, the media was changed to 2 mL DMEM, supplemented with 5% FBS, and the cells were cultured at 37 °C in a 5% (*v/v*) CO_2_ incubator. After 96 h, the supernatant was collected, filtered with a 0.45 μm filter, and used to infect BHK-21 cells. BHK-21 cells, grown overnight in 12-well plates to 70–80% confluency, were infected with 500 µL of rVSV-GFP or rVSV-mIL12-mGMCSF from the rescue step. One and a half hours later, 500 µL of fresh DMEM (containing 2% FBS and 2 mM L-glutamine) was added. GFP expression was detected by inverted fluorescence microscopy (Carl Zeiss Microscopy GmbH, Jena, Germany) 24 h later. The supernatants were collected after 72 h, centrifuged at 3000× *g* for 10 min at 4 °C, and filtered through a 0.45 μm membrane.

For concentration with the use of a sucrose cushion, 4 mL of a 20% sucrose cushion was overlaid onto the virus supernatant. The samples were centrifuged for 1 h at 120,000× *g* at 4 °C. The supernatant was discarded, and the virus pellets were resuspended in 1 mM Tris-HCl (pH 7.5), 1 mM EDTA, and 10% DMSO. Following a 1 h incubation, the sample was purified and concentrated in a sucrose gradient (25, 45, and 60%). The final viral preparation was then resuspended in PBS.

### 4.4. Median Tissue Culture Infectious Dose Assay

To determine the quantity of the infectious virus in supernatants, the TCID50 assay was performed as described previously [41]. BHK-21 cells were plated in 96-well plates, and 0.5 log serial dilutions of the virus were added to the wells 24 h later. After 72 h, the cells were examined to determine CPE. Final titers (TCID50/mL) were obtained using the Reed–Muench formula.

### 4.5. Assessment of IL-12 Activity in HEK-Blue IL-12 Cells

A 20 μL volume of diluted cytokine control (rIL-12 at 10 ng/mL) or supernatants containing rVSV-mIL12-mGMCSF amplified in two different volumes (T25 and T175 flasks) was added to 50,000 HEK-Blue IL-12 cells seeded in 180 μL of complete HEK-Blue assay media in a 96-well, flat-bottom plate. The plate was incubated for 16 h at 37 °C and 5% CO_2_. To assess IL-12 activity, the levels of SEAP expression were quantified using a QUANTI-Blue detection reagent (InvivoGen, San Diego, CA, USA), which was prepared as per the manufacturer’s instructions. A 180 μL volume of QUANTI-Blue detection reagent was added to 20 μL of culture supernatant and incubated for 3 h at 37 °C and 5% CO_2_. Absorbance was measured using a ClarioStar plate reader at 650 nm.

### 4.6. Transmission Electron Microscopy

The detection of viral particles in supernatants was carried out by TEM. A 10–20 µL volume of viral supernatant was applied to freshly glow-discharged copper grids (230 mesh, formvar-carbon coated) for 5 min, washed, and stained with 1 droplet of a 0.2% water solution of uranyl acetate. The grids were prepared in duplicates. The grids were observed with a transmission electron microscope (Carl Zeiss CrossBeam 550, Carl Zeiss Microscopy GmbH, Jena, Germany) operating at 80 kV. At least 10 grid squares were examined thoroughly, and representative micrographs were taken at different magnifications.

### 4.7. Confocal Microscopy

Confocal fluorescence images were obtained using an inverted point scanning confocal microscope (LSM 980 Airyscan based on Axio Observer 7, Carl Zeiss Microscopy GmbH, Jena, Germany) equipped with a motorized piezo stage through a 10× objective lens (EC Plan-Neofluar, numerical aperture 0.3, Carl Zeiss Microscopy GmbH, Jena, Germany). Images were obtained in Airyscan mode. rVSV-GFP was excited by a 488 nm laser diode (maximum power: 13 mW; AOTF transmission was set to 0.9%). Emission light was passed through the emission filter of the Airyscan (495–550 nm). Detector gain was set to 850 V. Confocal scan zoom was set to 2×, and image size was set to 1045 × 1045 pixels. The image parameters were set as follows: pixel dwell time, 1.96 micro s (scan speed 6); pixel size, dx = dy = 0.39 µm. Raw Airyscan images were processed by the Airyscan processing algorithm (processing strength 3.9). Images were obtained using Zen software (version Zen Blue 3.2, Carl Zeiss Microscopy GmbH, Jena, Germany).

### 4.8. In Vivo Studies

Male C57Bl/6 mice with a maximum age of 12 weeks were purchased from the SPF Animal Facility of the Institute of Cytology and Genetics, SB RAS (Novosibirsk, Russia). All animals were housed in standard polypropylene cages at a controlled temperature (25 ± 2 °C), light (12 h of light and dark cycle) and relative humidity (65 ± 5%) conditions. The animals were provided food and water ad libitum.

Approximately 1 × 10^6^ murine melanoma B16-F10 cells in 200 μL PBS were subcutaneously injected into the left flank. Tumor sizes were measured every 2 days with a caliper, and the volume was calculated using the formula length × width × height. When the mean tumor volume reached 500 mm^3^, the mice were intratumorally injected with rVSV or placebo control twice, with an interval of 3 days. Tumor sizes were measured every 2 days. The tumor growth inhibition index (TGII) was calculated using the formula (Vc − Vt)/Vc × 100, where Vc is the average volume of untreated tumors and Vt is the average volume of treated tumors. Moribund mice were sacrificed by cervical dislocation.

### 4.9. Statistical Analysis

The statistical analysis for the in vitro test was carried out using ordinary one-way ANOVA and Sidak’s multiple comparison test in GraphPad Prism. The statistical analysis for in vivo experiments was performed using Statistica 10.0 (Statsoft, Tulsa, OK, USA), and the graphical visualization was generated using SigmaPlot 12.5 (Systat Software Inc., San Jose, CA, USA). Values are given as mean ± SE; statistical significance was determined using a non-parametric, Mann–Whitney U-test. Mouse survival curves were plotted as Kaplan–Meier analyses. *p* < 0.05 was regarded as significant.

## Data Availability

The data presented in this study are contained within the article and available upon request from the corresponding author.

## Supplementary Materials

The following supporting information can be downloaded at: https://www.mdpi.com/article/10.3390/ijms25010211/s1.
