# Supplementary material for: Development of Recombinant Oncolytic rVSV-mIL12-mGMCSF for Cancer Immunotherapy
Source: Int J Mol Sci. 2023 Dec 22;25(1):211. doi: 10.3390/ijms25010211 (PMC10779112; doi:10.3390/ijms25010211)
Supplement: Supplementary file 1 [file ijms-25-00211-s001.zip › Supplementary Figure S1.pdf]

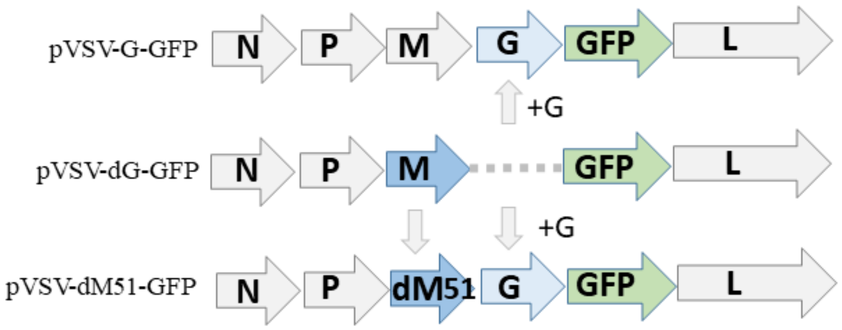

Supplementary Figure S1. Construction of pVSV-G-GFP and pVSV-dM51-GFP plasmids. The VSV glycoprotein gene (G) was PCR-amplified using pBS-G as a template and cloned into pVSV-dG-GFP lacking G gene. The resultant plasmid pVSV-G-GFP, containing the wild type sequence of M gene, was used as a template to introduce methionine deletion in position 51 (dM51). The resultant plasmid pVSV-dM51-GFP, similar to pVSV-G-GFP, encoded five viral proteins (N, P, G, M and L) and GFP reporter, with the exception of the dM51 deletion.
