# Supplementary material for: Development of Recombinant Oncolytic rVSV-mIL12-mGMCSF for Cancer Immunotherapy
Source: Int J Mol Sci. 2023 Dec 22;25(1):211. doi: 10.3390/ijms25010211 (PMC10779112; doi:10.3390/ijms25010211)
Supplement: Supplementary file 1 [file ijms-25-00211-s001.zip › Supplementary Figure S2.pdf]

METDTLLLWVLLLWVPGSTGAGGKPSGGRVIPVSGPARCLSQSRNLLKTTDDMVKTAREKLKHY
SCTAEDIDHEDITRDQTSTLKTCLPLELHKNESCLATRETSSTTRGSCLPPQKTSLMMTLCLGS
IYEDLKMYQTEFQAINAALQNHNHQQIILDKGMLVAIDELMQSLNHNGETLRQKPPVGEADPYR
VKMKLCILLHAFSTRVVTINRVMGYLSSGGGGSGGGGSGGGGSMWELEKDVYVVEVDWTPDAPG
ETVNLTCDTPEEDDITWTSDQRHGVIGSGKTLTITVKEFLDAGQYTCHKGGETLSHSHLLLHKK
ENGIWSTEILKNFKNKTFLKCEAPNYSGRFTCSWLVQRNMDLKFNIKSSSSSPDSRAVTCGMAS
LSAEKVTLDQRDYEKYSVSCQEDVTCPTAEETLPIELALEARQQNKYENYSTSFFIRDIIKPDP
PKNLQMKPLKNSQVEVSWEYPDSWSTPHSYFSLKFFVRIQRKKEKMKETEEGCNQKGAFLVEKT
STEVQCKGGNVCVQAQDRYYNSSCSKWACVPCRVRSGGGGSGGGGSAPTRSPITVTRPWKHVEA
IKEALNLLDDMPVTLNEEVEVVSNEFSFKKLTCVQTRLKIFEQGLRGNFTKLKGALNMTASYYQ
TYCPPTPETDCETQVTTYADFIDSLKTFLTDIPFECKKPGQKA

Supplementary Figure S2. The amino acid sequence of mIL12-mGMCSF fusion used in this study. Signal peptide is underlined, the linker connecting the fused proteins is in blue.
