# Supplementary material for: Development of Recombinant Oncolytic rVSV-mIL12-mGMCSF for Cancer Immunotherapy
Source: Int J Mol Sci. 2023 Dec 22;25(1):211. doi: 10.3390/ijms25010211 (PMC10779112; doi:10.3390/ijms25010211)
Supplement: Supplementary file 1 [file ijms-25-00211-s001.zip › Supplementary Table S1.pdf]

Name	Sequence (5′-3′)
prim#1	CTGTTTACGCGTCACTATGAAGTGCCTTTTGTACTTAGCC
prim#2	GCGAGTTGCTAGCAATTAATTCTC
prim#3	GACACCTATGATCCGAATCAATTAAGATATGAGA
prim#4	CTCGTCAACTCCAAAATAGGATTTGTCAATTGGA
prim#5	GCAAAGAATTCGCCGCCACCATGGAAGTCCACGATTTTGAGACC
prim#6	GAGGAGTGCGGCCGCTTAATCTCTCCAAGAGTTTTCCTCGTG
prim#7	GCAAAGAATTCGCCGCCACCATGGATAATCTCACAAAAGTTCG
prim#8	GAGGAGTGCGGCCGCCTACAGAGAATATTTGACTCTCG
prim#9	GCAAAGAATTCGCCGCCACCATGTCTGTTACAGTCAAGAGAATC
prim#10	GAGGAGTGCGGCCGCTCATTTGTCAAATTCTGACTTAGC
prim#11	GCGGCTAGCGACATGGAGACAGACACCCTCCTGC
prim#12	GCGCCTAGGTTATGCCTTCTGCCCTGGTTTCTTGC

Supplementary Table S1. Cloning and mutagenesis primers used to construct plasmids in this study. Restriction sites are underlined.
